# The applicability of species sensitivity distributions to the development of generic doses for phytosanitary irradiation

**DOI:** 10.1038/s41598-023-29492-1

**Published:** 2023-02-09

**Authors:** Cory Penca, Andrea L. Beam, Woodward D. Bailey

**Affiliations:** USDA-APHIS-PPQ-S&T Treatment and Inspection Methods Laboratory, Miami, FL USA

**Keywords:** Invasive species, Entomology

## Abstract

Ionizing radiation is used as a phytosanitary treatment to prevent the introduction of pests through trade. Generic doses are a valuable means to increase the number of pest-commodity combinations that can be treated using phytosanitary irradiation. Generic doses allow for the treatment of the entire taxa for which the dose has been approved, allowing for the treatment of untested species. As such, the approval of a generic dose requires substantial supporting data and careful consideration of the risks involved. We adopt the Species Sensitivity Distribution (SSD) framework, already in widespread use in the field of ecotoxicology and environmental risk assessment, to evaluate generic doses for phytosanitary irradiation treatments. Parametric SSDs for Curculionidae and Tephritidae were developed using existing data on efficacious phytosanitary irradiation treatments. The resulting SSDs provided estimates of the taxa coverage expected by the generic dose, along with the margin of uncertainty. The SSD analysis lends support to the existing 150 Gy generic dose for Tephritidae and a proposed 175 Gy generic dose for Curculionidae. The quantitative estimates of risk produced by the SSD approach can be a valuable tool for phytosanitary rule making, improving the process for generic dose development and approval.

## Introduction

The introduction of invasive species through global trade represents one of the most significant threats to global biodiversity and ecosystem services and is responsible for billions of dollars in economic losses to the agricultural sector through direct crop losses, increased management costs and loss of market access^1–5^. Phytosanitary treatments are an invaluable means of preventing the introduction of invasive species while facilitating global trade^6^. For established treatment technologies, such as methyl bromide fumigation, the acceptance of current phytosanitary treatment schedules is based on a combination of supporting research and a history of effective use. For newer treatment technologies, which lack a track record of efficacy, a substantial body of supporting research is required before acceptance by a National Plant Protection Organization (NPPO) or appropriate regulatory authority. Supporting research includes strong evidence of species-specific treatment efficacy, and in many cases the approval of the new treatment will be limited to the specific pest-commodity combinations where testing has occurred. For a single-species treatment schedule to be approved most regulatory authorities require a large sample size be tested without treatment failure. Historically this could require up to 93,613 individual insects being treated, as is the case for proving at least probit-9 level efficacy^7^. Arguments have been made that probit-9 criteria may be excessive for some pests, and methods for approving treatments that require smaller sample sizes have been proposed^8–10^. Nonetheless, widespread adoption of newer treatment technologies is limited in part by the lack of approved treatment schedules caused by the difficulties associated with building a body of supporting research to defend their use. Therefore, older technologies like methyl bromide fumigation are still widely used and their replacement has been slow.

Generic doses have been used by NPPOs, including the United States Department of Agriculture, Animal and Plant Health Inspection Service’s Plant Protection and Quarantine program (USDA-APHIS-PPQ), to increase the number of pest-commodity combinations for which a treatment can be used. A generic dose is a treatment schedule considered by researchers and/or regulatory authorities to be effective at mitigating all members of a specified taxon. In essence, generic doses increase the potential customer base for the phytosanitary treatment, providing an economic incentive for the wider adoption of the treatment technology. Generic doses offer an additional advantage when a new pest species is detected in a country. Such a discovery could disrupt trade until research is conducted to determine a treatment schedule for the new pest. However, if a generic treatment is available which covers the new pest, trade can continue with minimal or no disruption^11^.

The current method for establishing a generic dose for a particular taxon typically involves reviewing the literature and identifying relevant studies where the treatment has been applied to the taxon in question. A subjective judgement is then made as to whether enough members of the taxon have been tested and then a treatment dose at or near the dose required to control the most tolerant member of the taxon is selected to serve as the generic dose. Whether the generic dose is likely to cover species not represented by the literature review is unknown and criteria are not established for the number of species in a taxon which must be studied before a generic dose can be approved for that group. In general, if data are present for the majority of the most economically important or quarantine significant species then there is confidence that the generic dose will be secure enough for phytosanitary use. This general approach has been used in proposing generic irradiation treatments at the genus level (i.e. *Anastrepha* ), the family level, as in Tephritidae^12,13^ and Tortricidae^14^, the level of order for Lepidoptera^15^, and broadly for the class Insecta excluding pupal and adult Lepidoptera^16^. Generic doses for these taxa are included in the APHIS-PPQ Treatment Manual and the generic doses for *Anastrepha*, Tephritidae and Tortricidae have been accepted by the International Plant Protection Convention’s Commission for Phytosanitary Measures and have been adopted under ISPM 28: Phytosanitary treatments for regulated pests^17,18^.

A major drawback to current ‘subjective’ approaches to generic dose setting is that there is a lack of information on what portion of the taxa might require a dose higher than the generic dose. Having an estimate of the taxonomic coverage of the generic dose will provide policy makers with a quantification of the potential risk that a member of the taxa will not be controlled by the treatment. A possible means to achieve a quantitative estimate of the taxonomic coverage of a generic dose is via the species sensitivity distribution (SSD) method, a widespread approach in ecotoxicology^19^. The SSD method looks at the distribution of toxicity values for multiple species, and after fitting these values to an appropriate distribution (as in the parametric approach), estimates the percentile of species affected at varying doses/concentrations from the resulting cumulative distribution function^20^. Species sensitivity distributions have been used to set environmental limits for a variety of toxicants, most frequently in aquatic environments. The SSD approach has been adopted in various forms by environmental regulatory agencies in numerous countries^19,21^. The United States Environmental Protection Agency has developed a web-accessible “SSD Toolbox” to assist with conducting and interpreting SSD analysis for environmental toxicants (EPA 2020), whereas Australia and New Zealand have co-developed a software tool called “Burrlioz” for similar purposes^22^. A key advantage of the SSD approach is the ability to extrapolate beyond the available data and provide estimates of uncertainty^23^. We introduce the SSD method as a tool for evaluating generic doses for phytosanitary treatments with a demonstration using reported phytosanitary irradiation doses for the dipteran family Tephritidae and the coleopteran family Curculionidae. We conducted the SSD approach by using available data for Curculionidae and Tephritidae, along with an interval-censored SSD to adjust for differences in data quality presented in the Curculionidae source studies. We address the challenges to the successful application of the SSD approach, referring to the body of ecotoxicology literature that has been generated in an attempt to address these challenges. Specifically, we address concerns regarding data quality in the source studies and the challenges of correctly interpreting the results of an SSD analysis when setting the minimum dose in a phytosanitary treatment schedule. Lastly, we provide recommendations for future phytosanitary irradiation research that accommodates and incorporates the use of the SSD approach to generic dose setting for phytosanitary treatments.

## Results

### Curculionidae crude SSD

A total of 28 species of Curculionidae from 20 genera were identified where studies on the irradiation dose required for prevention of F1 development to adults were present in the literature (Table 1). Our Curculionidae literature review expands on those of Hallman^24^ (14 species) and Follett^25^ (15 species) who have proposed a generic dose of 150 Gy for this family. The highest reported dose in our literature review was 250 Gy, reported for *Naupactus xanthographus.* This dose appeared to be arbitrarily selected (no doses below 250 Gy were tested) and was identified as an extreme outlier (Cook’s distance greater than 4/*n*). This dose was removed from the dataset used for the calculation of the SSD.

**Table 1:** Efficacious doses from Curculionidae literature review.

Species	Minimum effective dose	Specimens treated	Reference
*Anthonomus grandis*	100	120	Earle et al.^26^
*Blosyrus asellus*	25	15	Follett et al.^27^
*Conotrachelus nenuphar*	92	25,000	Hallman^28^
*“ “*	80	8	Jacklin et al.^29^
*Cryphalus fulvus*	150	50	Yoshida et al.^30^
*Cylas formicarius elegantulus*	150	62,623	Follett^31^
*Diaprepes abbreviatus*	50	220	Gould & Hallman^32^
*Euscepes postfasciatus*	150	62,323	Follett^31^
*Hylurgus ligniperda*	175	60	van Haandel et al.^33^
*Hypera postica*	80	30	Burgess & Bennett^34^
*Hypothenemus hampei*	100	6,598	Follett^25^
*“ “*	75	138	Follett^25^
*Ips confusus*	150	30	Wood & Stark^35^
*Ips sexdentatus*	140	–	Wang et al.^36^
*Ips subelongatus*	140	–	Zhan et al.^37^
*Naupactus xanthographus*	250	60	Gerstle & Sazo^38^
*Orchidophilus aterrimus*	150	10	Manoto et al.^39^
*Phlyctinus callosus*	80	200	Duvenhage & Johnson^40^
*Pissodes strobi*	44	40	Jaynes & Godwin^41^
*Sitophilus granarius*	100	80	Aldryhim & Adam^42^
*Sitophilus oryzae*	120	32,025	Follett et al.^43^
*Sitophilus zeamais*	70	280	Hu et al.^44^
*Sphenophorus levis*	25	100	Arthur & Wiendl^45^
*Sternochetus frigidus*	109	1,480	Obra et al.^46^
*Sternochetus mangiferae*	100	76	Follett
*“”*	50	60	Seo et al.^47^
*Xyleborus atratus*	50	12	Yoshida et al.^48^
*Xyleborus perforans*	50	12	Yoshida et al.^48^
*Xylosandrus compactus*	40	12	Yoshida et al.^49^
*Xylosandrus crassiusculus*	40	12	Yoshida et al.^49^
*Xylosandrus germanus*	40	12	Yoshida et al.^49^

**Figure 1:**
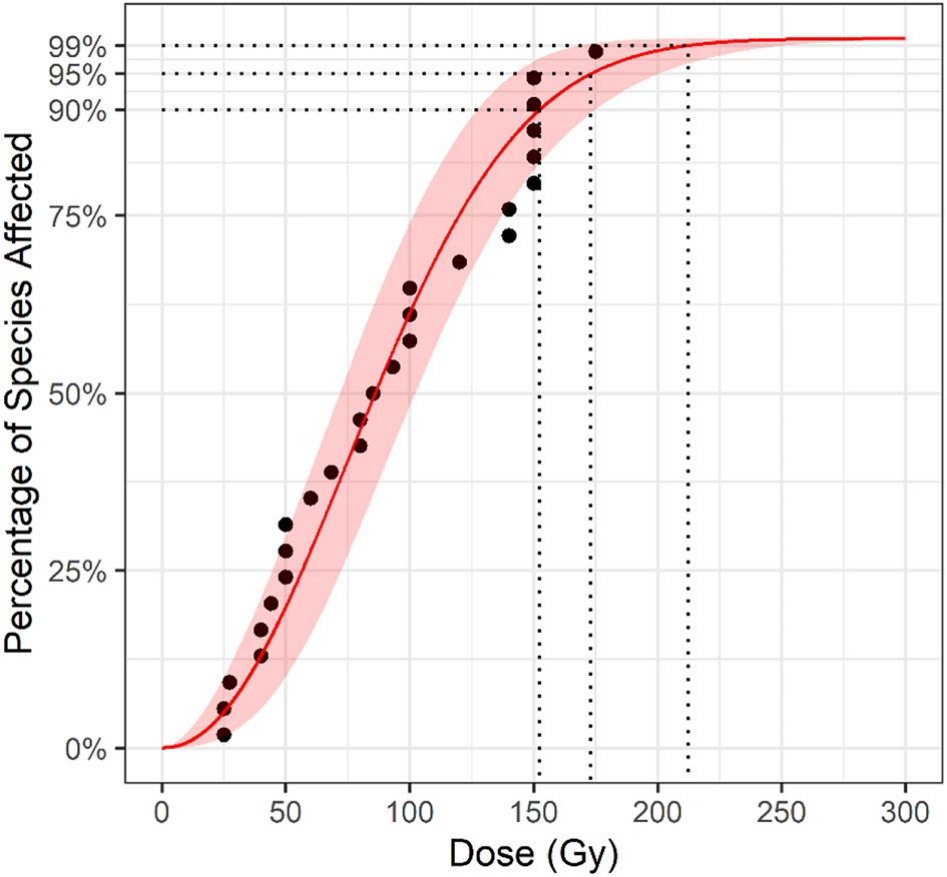
SSD Curve for Curculionidae fitted to the Weibull distribution with the shaded area covering the 5–95% confidence interval.

**Figure 2:**
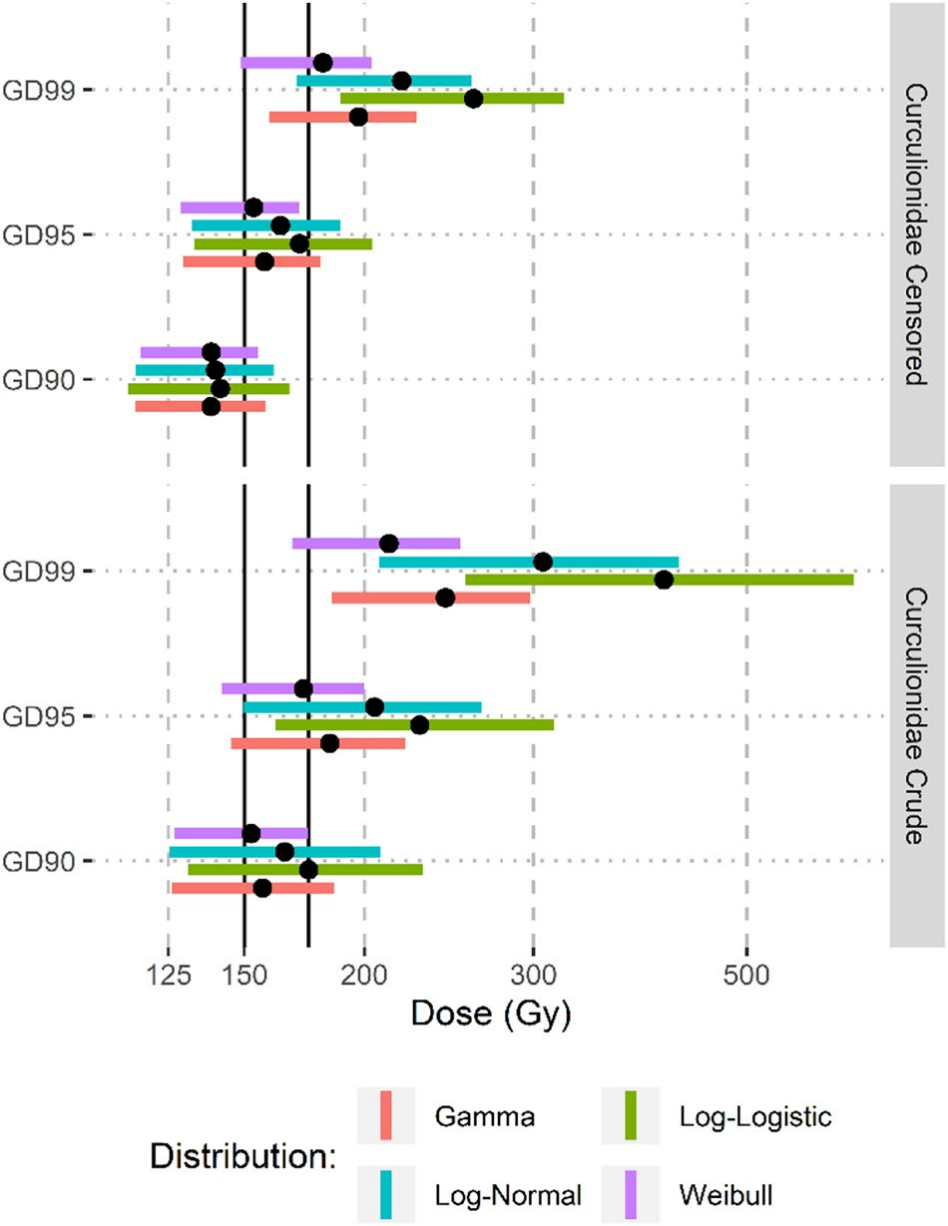
Point estimates and the lower 5% to upper 95% confidence intervals (colored bars) for the generic irradiation dose required to obtain 90, 95 and 99 percent coverage of the family Curculionidae.

The Weibull distribution showed the best fit for the Curculionidae data based on AIC, however the differences between distributions were small (Table 2). The resulting SSD curve for Curculionidae is presented in Fig. 1. The GD90 and GD95 estimates (i.e., the generic doses predicted to control 90% and 95% of the taxa) for the Weibull distribution were both lower than 175 Gy, with the 90% CI for the GD90 estimate falling entirely below 175 Gy for the Weibull distribution (Fig. 2). At a dose of 175 Gy the estimated species coverage was 95.4% (90% CI: 90.2–99.4%). In comparison, the proposed 150 Gy generic dose provides an estimated species coverage of 89.2% (90% CI: 82.0–96.8%).

**Table 2:** Model Goodness-of-Fit Test Results.

Data		Distribution			
	Measure	Gamma	Log-Logistic	Log-Normal	Weibull
Curculionidae (crude)	AD	0.77	0.84	0.86	0.76*
	AIC	285.16	288.96	286.92	284.18*
Curculionidae (censored)^a^	AIC	62.2	63.8	62.1*	63.1
Tephritidae (crude)	AD	0.28*	0.30	0.37	0.29
	AIC	151.41*	152.42	152.22	151.55

^a^Anderson-Darling statistic not computed for censored data. *AD* Anderson–Darling statistic, *AIC* Akaike’s information criterion. *Indicates the best fitting model.

### Curculionidae Interval Censored SSD

**Figure 3:**
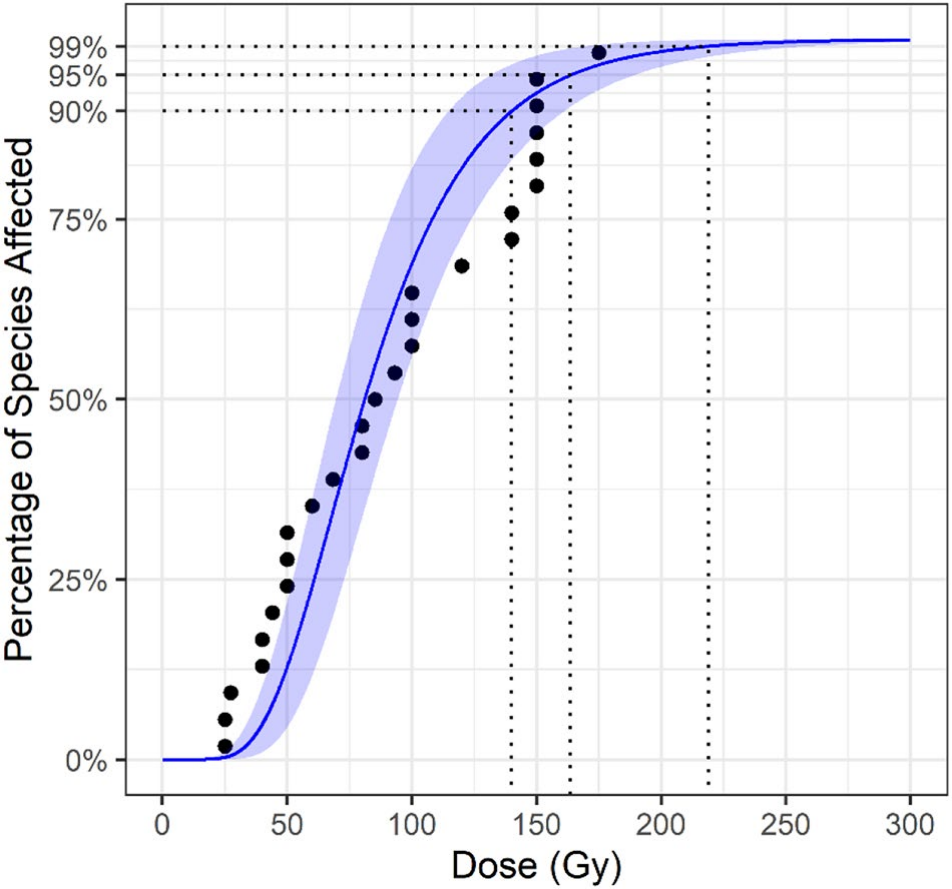
SSD Curve for Curculionidae using interval-censored data with the shaded area covering the 5–95% confidence interval. Points are fitted to the log-normal distribution.

**Figure 4:**
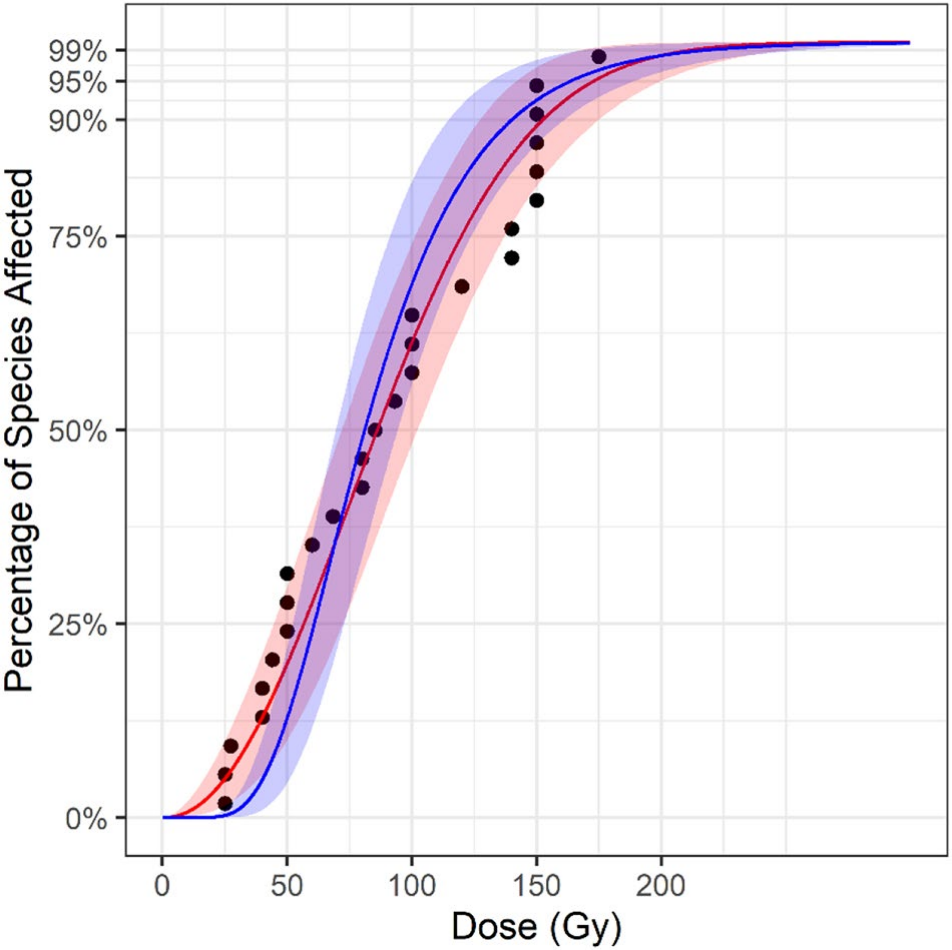
Comparison of the Crude SSD (red) and Interval Censored SSD (blue) for Curculionidae.

The lognormal distribution provided the best fit for the interval censored dataset. The resulting SSD curve from the interval censored data was steeper than the SSD curve resulting from the crude analysis (Fig. 3). Estimated coverage at the range of interest was increased when using the interval censored SSD and the point estimates converged around 190 Gy (Fig. 4). At 175 Gy, the estimated species coverage for the interval censored SSD was 96.4% (90% CI: 93.0–99.3%) while the proposed 150 Gy generic dose provides an estimated species coverage of 92.5% (90%CI: 87.1–98.0%). The interval censored – lognormal estimate for the GD90 was 140.08 Gy (90% CI: 115.74–161.02), for the GD95 the estimate was 163.59 Gy (90% CI: 132.37—189.04) and for the GD99 the estimate was 218.84 Gy (90% CI: 170.10–258.54) (Fig. 2). When interval-censored estimates were compared to crude estimates from the same distributions the interval-censored estimates were lower, ranging from a 13- 33 Gy difference at the GD90 coverage level to a 31- 150 Gy difference at the GD99 coverage level, with the largest differences between crude and interval censored estimates occurring in the log-logistic SSD (Fig. 2).

### Tephritidae crude SSD

A total of 15 species of Tephritidae were identified where research has been conducted on the ability of irradiation to prevent late instar larvae from developing to adults. For several species multiple studies had been conducted and were combined into a single dose for analysis. Table 3 presents the combined doses used to generate SSD curves.

**Table 3:** Sterilizing doses from Tephritidae literature review. Data from multiple studies are combined for each species. End point was prevention of emergence of normal-looking adults.

Species	Dose Estimate^a^
*Anastrepha fraterculus*	41.48
*Anastrepha ludens*	66.58
*Anastrepha obliqua*	59.78
*Anastrepha serpentina*	60
*Anastrepha striata*	40
*Anastrepha suspensa*	51.13
*Bactrocera cucurbitae*	144
*Bactrocera dorsalis*	116
*Bactrocera* near* dorsalis*	100
*Bactrocera jarvisi*	101
*Bactrocera tryoni*	86.26
*Bactrocera zonata*	55
*Ceratitis capitata*	114.21
*Rhagoletis indifferens*	18
*Rhagoletis pomonella*	20

^a^Estimate taken as the geometric mean of reported doses, with the maximum dose set as the lowest dose where the confidence in 99.99% efficacy was > 0.99.

**Figure 5:**
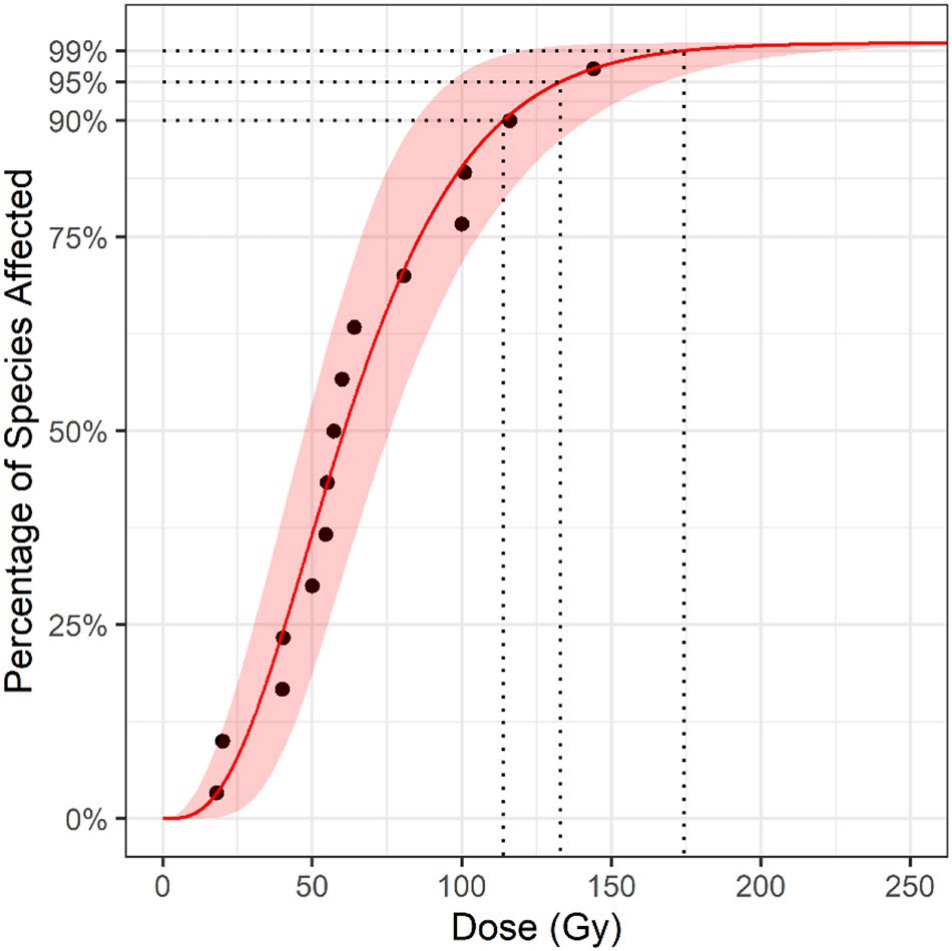
SSD Curve for Tephritidae with the shaded area covering the 5–95% confidence interval. Points are fitted to the gamma distribution.

**Figure 6:**
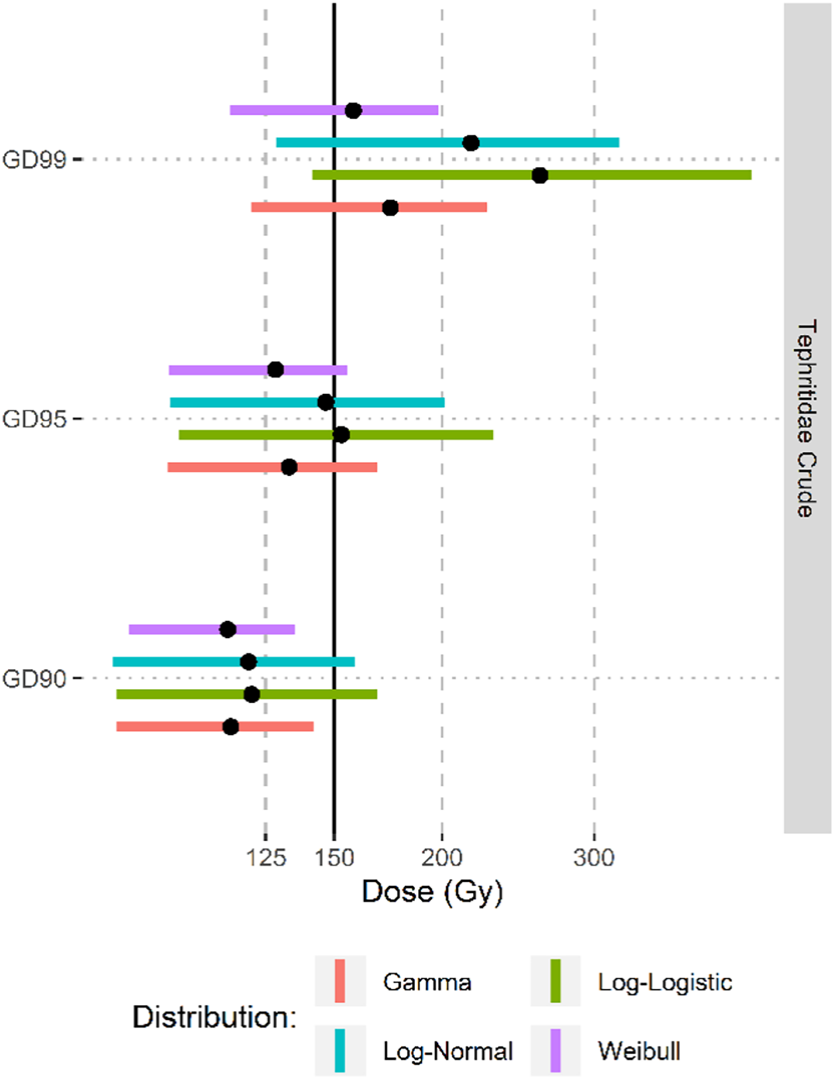
Point estimates and the lower 5% to upper 95% confidence intervals (colored bars) for the generic irradiation dose required to obtain 90, 95 and 99 percent coverage of the family Tephritidae.

The gamma distribution provided the best fit for the Tephritidae data (Table 2). The resulting SSD curve is presented in Fig. 5. The GD90, GD95 and GD99 were estimated at 113.88 (90% CI: 84.07–141.86), 133.08 (90% CI: 96.18–168.13), 174.30 (90% CI: 120.21–225.19) respectively (Fig. 6). The U.S. Department of Agriculture (USDA)-approved generic dose of 150 Gy was estimated to cover 97.4% of species in Tephritidae (90 %CI: 92.1 – 99.9).

### Tephritidae small sample size bias correction

**Figure 7:**
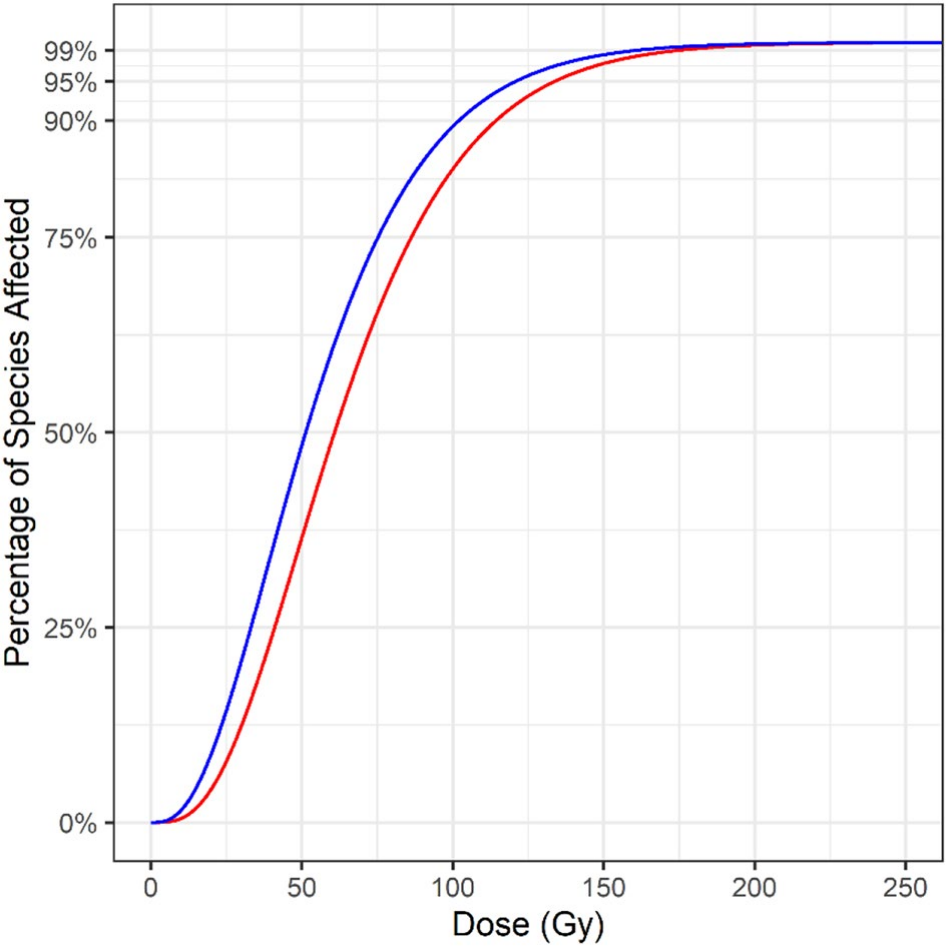
Comparison between the small sample size bias-corrected SSD (blue) and the crude SSD (red). The leftward shift indicates that increasing the number of Tephritidae species used in the SSD model will result in a reduction in the required generic dose.

When the Cox-Snell method for small sample size bias correction was applied to the Tephritidae dataset, the resulting SSD curve was shifted leftward with the estimates for the GD90, GD95 and GD99 values being 12.29, 12.93, and 13.93 Gy lower, respectively, than the estimates from the gamma distributed crude SSD (Fig. 7). At the 150 Gy approved generic dose the sample size bias corrected SSD provided 1.11% greater coverage than the gamma distributed crude SSD.

## Discussion

The present study provides an example of how species sensitivity distributions can be applied to generic doses for phytosanitary treatments, providing a valuable quantification of the expected taxonomic coverage of the generic dose. This technique could be widely applicable and assist the international phytosanitary community in making decisions about adopting new phytosanitary treatments. The desired level of coverage necessary for a generic phytosanitary irradiation treatment is not resolved and may ultimately be determined be each country based on an assessment of acceptable level of risk. In this work we presented results at 3 levels of coverage (GD90, GD95, GD99), however the actual level of coverage required should be based on a consideration of various risk factors associated with the taxa and commodity of interest, including infestation rates, consignment sizes, establishment probabilities and the impact of establishment of the major pests within the taxa. Coverage greater than 95% (GD95) was obtained for Tephritidae at the current APHIS-approved generic dose of 150 Gy, and for Curculionidae at the proposed generic dose of 175 Gy. Whether coverage at the GD95 level is sufficient requires careful consideration. In the case of both Tephritidae and Curculionidae the generic doses were developed using data that included several of the most important pest species in each taxa and the recommended doses are above the required dose for the most radiotolerant of these pest species. Furthermore, the majority of species in both taxa are rarely (or never) encountered in trade and are not considered important agricultural or environmental pests. Thus, it is unlikely that a species in the < 5% of the taxa not covered by the generic dose will be consequential in the phytosanitary treatment context.

In practice phytosanitary irradiation treatments do not deliver precise doses; rather a range of absorbed doses will be experienced, spanning the minimum dose (Dmin) up to the maximum dose (Dmax). The dose uniformity ratio for an irradiation treatment is equal to Dmax/Dmin and can vary between treatment operators depending on commodity density, process configuration and irradiation source. In order for a phytosanitary irradiation treatment to be considered successful the measured Dmin value must not fall below the dose required in the approved treatment schedule. The actual dose received by a pest may be 20–100% greater than the required dose depending on the DUR for that treatment. The increased dose absorbed due to the DUR provides an additional level of protection beyond what is predicted by the SSD method, thus the estimates given by the SSD method can be considered conservative in practice. If the required treatment dose to obtain sufficient coverage is not feasible due to damage to the commodity, then phytosanitary irradiation may not be an appropriate treatment for that commodity. It may also prove desirable to increase the generic dose until the slope of the SSD falls below a value *m* (i.e., for every 1 Gy increase in dose a *m*% increase in coverage is obtained). This approach implies a trade-off between the cost of increasing the minimum dose requirement and the reduction of risk obtained from increasing taxa coverage. The nature of this relationship, and the value for *m,* should be defined. Following this method when an arbitrary *m* value of 0.1% is used, then the suggested generic dose for Curculionidae derived from the censored SSD would be 177 Gy, beyond which the gain in coverage per unit increase in dose is less than 0.1%. A dose of 177 Gy is estimated to be effective to control 96.6% of species in Curculionidae.

Data quality plays an important role in producing accurate estimates from SSD models. While a number of factors influence the perceived quality of the estimates produced by the studies used to develop the SSD model, in phytosanitary irradiation research two primary factors help differentiate data quality. The first is the presence of testing at multiple doses below the recommended dose. Under an ideal design these results can be analyzed by regression to produce an estimate at a given level of efficacy along with a confidence interval for that estimate. Even in studies where a dose–response analysis was not conducted, having multiple doses tested below the recommended treatment dose helps limit the magnitude of an overestimate. The second data quality factor is the number of specimens tested at the recommended dose. In phytosanitary research this is referred to as confirmatory testing, and the current standard is to test upwards of 30,000 (probit 8.72) to 93,613 (probit 9) individuals with no survivors. Increasing the number of individuals tested at the recommended dose will prevent underestimating the phytosanitary dose. For example, if 50 Gy was the probit 6-equivalent dose (84.13% efficacy at the 95% confidence level), and only 12 specimens were tested, the probability of observing no survivors is approximately 12.5%, whereas if 200 specimens were tested, the probability of observing no survivors is less than 0.00003%. The efficacy at a given dose is not known a priori*,* however as efficacy increases with dose, it follows that higher values of *n* at lower doses will prevent misleading observations of 100% efficacy at doses below the true phytosanitary dose. When treatment efficacy requirements are determined using a risk-based approach efficacy levels below 99.99% / probit-8.72 can be considered sufficient for phytosanitary treatments, as may occur in cases where the initial infestation rate is low due to poor host status of the commodity or effective management of the pest at the place of production^10,50^. In such cases an SSD-derived generic dose that aims to achieve 99.99% or greater efficacy for each species in the taxa may result in “overkill”, wherein the treatment is in excess of what would be required to reach the desired risk-based criteria for efficacy.

Our conclusion that a generic dose of 175 Gy can be expected to control 96.4% of Curculionidae contains several important caveats. The treatment end points included studies where F1 mortality occurred in the egg stage and also studies where F1 mortality occurred after eclosion but none of the F1 generation developed to the adult stage. The doses required to prevent egg hatch and the doses required to prevent F1 larvae from reaching the adult stage are not equivalent, and this complicates direct comparisons. This highlights the importance of using consistent endpoints in generic dose research. We believe the source data includes an inherent bias towards conservative (higher) doses. This will result in shifting the SSD curve to the right, resulting in underestimate coverage at proposed doses. Underestimating the required dose, as occurs when too few specimens are tested, can also impact our model by increasing the variance around the mean. Our source data included several reported 40 Gy doses, conducted with only 12 insects^48,49^. These data points likely do not represent a true phytosanitary dose. The shifting of the curve at the extremes can influence distribution selected for the SSD model and ultimately the dose estimates for GD95. When the issue of data quality and variability in the confidence in the reported doses was addressed using interval censored data, we observed a downward shift in the doses required to obtain taxonomic coverage at GD90, 95 and 99.

A key assumption when using SSDs is that the source data is unbiased for the group being observed. In our example, the species selected from Curculionidae and Tephritidae are decidedly biased; they are largely the economically important species detected in trade. For example, 6 of the 10 most frequently intercepted Curculionidae genera reported in the USDA Pest ID database have been subject to phytosanitary irradiation research (Table 4). There may also be a bias towards genera which are amenable to laboratory rearing or field-capture in sufficient quantities for use in quarantine research. The need for an unbiased selection of species for proper application of the SSD method reduces the importance in testing specific species of economic importance. This may prove advantageous to phytosanitary treatment researchers, as often these species require special precautions, including working under quarantine conditions, or conducting the research in their native range. The SSD approach values testing more species from a greater taxonomic diversity but is agnostic with regards to the economic importance of those species. As such, researchers can conduct studies from members of the target taxa present in their area, making the research logistically simpler and allowing for more species to be tested. Methods of measuring taxonomic representatives of a sample abound^51,52^ and would be a valuable component for generating generic doses at higher taxonomic levels (i.e. an order level generic dose for Coleoptera). However, it is likely that National Plant Protection Organizations will still favor having testing done on the important economic species before adopting a generic dose.

**Table 4:** Frequently intercepted Curculionidae genera (2015–2019).

Genus	Specimens Intercepted (2015–2019)	Represented in Table 1
*Sitophilus*	3426	Yes
*Sternochetus*	1483	Yes
Curculionidae (family level ID)	1211	–
*Anthonomus*	671	Yes
*Curculio*	542	No
*Metamasius*	340	No
*Conotrachelus*	324	Yes
*Hypothenemus*	318	Yes
*Xyleborus*	293	Yes
*Sphenophorus*	232	No
*Balanogastris*	220	No

The sample size of 15 species used to produce the SSD for Tephritidae is considered marginally sufficient^53^, however the maximum likelihood estimator used to fit the data to a distribution is known to exhibit sample size bias. The Cox-Snell method for small sample size bias adjustment indicated that dose estimates are likely to decline if more species are included in the analyses. As both the censored estimates and the sample-size bias corrected estimates resulted in lower recommended generic doses there is evidence that the SSD approach will result in overestimates when data quality is poor or when the number of available studies is sparse. While efforts should be made to maximize the quality of data and increase the number of species studied, for the purpose of setting regulatory requirements our results suggest the crude SSD approach may result in conservative doses when data quality/quantity is sub-optimal.

As generic doses are produced through the analysis of research from a variety of sources, collaboration and cooperation between researchers is essential if the SSD approach is to be used to set and validate new generic doses. Specifically, the data being used to generate the SSD must be of similar quality or produced and reported in such a manner that differences in data quality (i.e., number of replicates, number of doses tested) can be accounted for in the SSD model. We recommend considering the following when undertaking research to support a generic treatment:

1. Specify the scope of the generic dose as accurately as possible (e.g., Genus, Family or Order)
2. Select species for testing which represent the scope as defined previously. The number of species should be sufficient to capture the taxonomic diversity within the group. We recommend a minimum of 10 species^54^, though 13 or more is preferable^53^. Economically important species should be included, but quantity and diversity may prove more important.
3. Identify a uniform end point and target lethality/efficacy (i.e., an ED99.99), ideally selected under a risk-based framework.
4. Produce data that includes the estimated dose to achieve the targeted level of efficacy and describes the error associated with the estimate. This requires testing doses that do not provide 100% efficacy. Regression or a comparable approach is preferred.
5. If confirmatory testing is to be done, select the lowest dose possible where 100% treatment efficacy is expected (avoid gross overestimation).

The advancements in the ecotoxicological application of species sensitivity distributions may have parallel applications for generic doses. For example, Duboudin et al.^55^ and Hiki & Iwasaki^56^ investigated whether acute SSDs can predict chronic SSDs^55,57^. A generic dose equivalent would be to ask whether lethal endpoint SSDs can predict sterility endpoint SSDs (or vice versa). Another example is the efforts to use phylogeny and species traits to make predictions about sensitivity to various toxicants^56,58–60^. Assuming phylogenetic relationships are reliably informative of radiotolerance, then a species whose susceptibility to phytosanitary irradiation is predicted to be lower than the generic dose generated by the SSD can be treated with a lower dose, providing savings and efficiencies to treatment operators. More generally, refinements to model selection, techniques for addressing data quality concerns, and the application of Bayesian methods can be applied to improve the reliability of generic dose estimates made using species sensitivity distributions^54,61–63^.

This work shows that species sensitivity distributions can be produced from phytosanitary irradiation data, with resulting estimates and error terms that align with expectations and current approved doses. Although this technique seems particularly well suited for developing generic irradiation treatments, it may not be as suitable for other treatment types. For example, for cold treatment, cold tolerance can vary by both insect species and by commodity for the same insect species^64–66^ and the models would need to account for multiple variables. Due to this additional factor, it may be more difficult to use SSD for developing generic cold treatments. The parametric SSD approach used in this study was derived from prior ecotoxicological work designed to determine the HC_5_ for chemicals in aquatic environments, and it must be acknowledged that the generic dose has significant differences with regards to the source data and the end purpose. As such, future efforts are needed to validate and optimize the methodology for applying SSD analysis to phytosanitary purposes, with the goal ultimately being an SSD approach for phytosanitary generic dose setting which stands independent of its ecotoxicological origins.

## Materials and methods

### Data sources

The reported doses used in our SSD analysis were obtained from a review of the relevant studies on phytosanitary irradiation of members of the Coleopteran family Curculionidae and the Dipteran family Tephritidae. These datasets are provided in the supplementary file, supplementary file. The studies used to generate the data set for Curculionidae were identified through a prior review of the literature conducted during an APHIS treatment approval request and are an expansion on prior reviews of phytosanitary irradiation of Curculionidae which had resulted in a recommended 150 Gy generic dose for this family^24,25^. Recorded doses for Curculionidae were limited to studies where the treatment endpoint was failure of F1 development at the reported dose (i.e., treated individuals may oviposit but eggs either fail to hatch or fail to develop into adults). The literature on Curculionidae irradiation included studies which used crosses of treated *x* untreated organisms, however for our purposes only crosses where both sexes were treated were included as this represents what is expected in a phytosanitary treatment where the entire consignment receives no less than the minimum required dose. Furthermore, when testing was conducted separately for male and female Curculionidae, we recorded the effective dose for females, as the treatment endpoint measured as failure to complete development of the F1 progeny produced by the female. Studies which focused on finding the sterilizing doses for males for the purpose of a sterile release program were deemed not suitable when the resulting level of sterility was less than 100%. This review resulted in phytosanitary radiation doses for 28 species. The lowest dose which resulted in 0 survivors, along with the number of individuals tested at that dose, was recorded for all studies which met our criteria and is presented in Table 1. Outliers were removed from the Curculionidae dataset used in the crude analysis based on Cook’s distance as determined by a linear model specified as *Reported dose* ~ *n* + *F,* where *n* is the number of specimens treated in the study and *F* is the highest dose where treatment failure was observed. Observations where Cook’s distance was greater than 4 times the mean were removed. This resulted in the removal of a single observation where the dose selected (250 Gy) appeared to be arbitrarily high, with only a single dose tested^38^.

The phytosanitary irradiation treatments for Tephritidae included in our analysis were restricted to those presented in Hallman & Loaharanu^12^ with the addition of data from Follett & Armstrong^13^ and Zhao et al.^67^. The treatment endpoint for Tephritidae was the failure of normal adult emergence from treated late instar larvae.

When multiple effective doses were presented for a species (usually resulting from independent studies) these data were combined to provide a single dose for that species. The combined dose was determined by taking the geometric mean of the reported doses for that species, weighted using the confidence of 99.99% efficacy where confidence was calculated as^7^:

1 $$C = 1 - \left( {1 - p} \right)^{n}$$

When multiple doses were reported in the literature where *C* was greater than 0.9999 only the lowest of these doses was retained.

### Curculionidae and tephritidae crude SSD analysis

Our approach to SSD curve fitting involved fitting the reported phytosanitary doses obtained from the literature review to four distributions; the log-normal, log-logistic, gamma and Weibull, through maximum likelihood estimation using the function ‘ssd_fit_dists’ in the R package ‘ssdtools’^68^. We refer to this analysis as the “crude” analysis as the doses obtained during the data collection phase were not adjusted outside the use of weighted averages for species with multiple reported doses. The distribution with the lowest Akaike’s information criterion (AIC) score, adjusted for sample size, was selected for further analysis^69^. The value for dose (*x*) of the fitted cumulative distribution function at *p* = 0.90, 0.95 and 0.99 along with 90% confidence intervals was calculated at each value of *p* from the resulting parametric bootstrap (5,000 iterations) of the distribution fit . This result is analogous to the HC_5_ values (hazardous concentration to 5% or less of species within the target community) in ecotoxicological studies, in which *p* = 0.05. In keeping with the SSD literature, we refer to the GD_p_ value as the generic dose in which taxonomic coverage is estimated to be *p*, expressed as either a proportion or a percent (i.e., a GD95 value implies the dose is effective for 95% of the taxon). In addition, the obtained treatment coverage, *p*, as a percent, when the dose was set at proposed generic doses of 150 and 175 Gy for Curculionidae^25^ and 150 Gy for Tephritidae^12^ was calculated using the ‘ssd_hp’ function in the R package ‘ssd_tools’.

### Interval censored SSD estimates for curculionidae

The quality of the data supporting the reported doses for Curculionidae in the literature was highly variable; the sample size *n* ranged from 8 to over 60,000 insects, while other studies lacked testing at multiple doses or doses below the reported dose of no observed progeny. The reported doses could thus be considered censored estimates of varying interval widths where the narrower the interval the more precise the estimate of the phytosanitary dose. We created balanced censored intervals such that, for the entire dataset, the mean difference between the lower end of the censored interval and the reported dose was approximately equal to the mean difference between the upper end of the censored interval and the reported dose. To do this we set the lower interval limit as the highest dose tested which resulted in less than 100% sterility, with the minimum value being 25 Gy, equivalent to the lowest effective dose reported in the literature*.* We then set the upper interval *U* for each reported dose *D* by adding the product of the confidence deficit and a factor *A* (Eq. 2), where the confidence deficit is calculated as 1-*C*, with *C* being the confidence in the reported dose being 99.99% effective (Eq. 1). The value for A was calculated using Eq. 3, where ${u}_{l}$ is the mean difference between the lower interval value and the reported dose *D* and ${u}_{c}$ is the mean confidence value $C$.

2 $$U = D + \left( {A*\left( {1 - C} \right)} \right)$$

3 $$A = \frac{{u_{l} }}{{1 - u_{c} }}$$

This approach for setting the upper interval reflects the fact that dose estimates produced from studies with large numbers of specimens tested represent the upper limit of what the true phytosanitary dose should be and that testing at sub-effective doses provides an estimate for the lower limit. The interval values were used for the SSD analysis following the same method as the “crude” analysis, with the additional specification of the intervals included when using the “ssd_fit_dists” function to determine the appropriate distribution for the SSD analysis. The values for GD90, GD95, and GD99, along with the expected coverage at 150 Gy and 175 Gy were calculated following the same method used for the “crude” analysis.

### Tephritidae small sample size bias correction

To demonstrate the influence of sample size bias on the generic dose estimates we used the Cox-Snell method for a gamma distribution (function “coxsnell.bc in the R package ‘mle.tools’) to correct for sample size bias 49,50. The resulting shape and scale parameters of the gamma distribution for the original model fit and the bias corrected fit were used to determine the dose required to achieve GD90, GD95 and GD99 coverage via the quantile function for the gamma distribution (function ‘qgamma’ in the R package ‘ssdtools’) with p set to 0.90, 0.95 and 0.99. The coverage at 150 Gy for the original model and bias corrected model were calculated using the R function ‘pgamma’ with q set to 150 Gy.

## Supplementary Information

Supplementary Information 1.

Supplementary Information 2.

## Data Availability

All data generated or analyzed during this study are included in this published article and its Supplementary Information files.
